# *rac*-*tert*-Butyl 2-{5-[(4-{2-[methyl(pyri­din-2-yl)amino]ethoxy}phenyl)methyl]-2,4-dioxo-1,3-thiazolidin-3-yl}acetate

**DOI:** 10.1107/S1600536811012177

**Published:** 2011-04-07

**Authors:** Sixing Hu, Guojuan Liang, Dashu Fang, Yongjun Gan, Xiangnan Hu

**Affiliations:** a College of Pharmaceutical Sciences, Chongqing Medical University, Chongqing 400016, People’s Republic of China

## Abstract

The title compound, C_24_H_29_N_3_O_5_S, is a chiral mol­ecule which crystallizes in a centrosymmetric space group as a racemate. The thia­zolidine ring forms the dihedral angles of 29.22 (12) and 67.79 (10)° with the benzene and pyridine rings, respectively. The benzene and pyridine rings are tilted by dihedral angle of 67.18 (9)°. In the crystal, inter­molecular C—H⋯O hydrogen bonds link the mol­ecules into a two-dimensional network.

## Related literature

For the related structure of 5-[[4-[2-(methyl-2-pyridinyl­amino)­eth­oxy]phen­yl]meth­yl]-2,4-thia­zolidinedione, see: Lei *et al.* (2003 ▶); Balint & Nagy (2006 ▶).
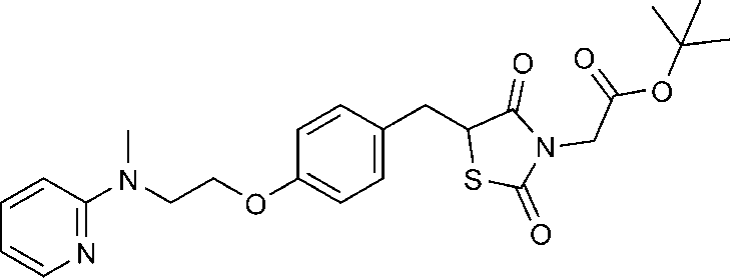


## Experimental

### 

#### Crystal data

C_24_H_29_N_3_O_5_S
*M* *_r_* = 471.56
Monoclinic, 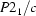

*a* = 25.621 (5) Å
*b* = 9.886 (2) Å
*c* = 9.874 (2) Å
β = 97.32 (3)°
*V* = 2480.6 (9) Å^3^
*Z* = 4
Mo *K*α radiation
μ = 0.17 mm^−1^
*T* = 291 K
0.40 × 0.13 × 0.12 mm

#### Data collection

Rigaku R-AXIS RAPID diffractometer
Absorption correction: multi-scan (*ABSCOR*; Higashi, 1995 ▶) *T*_min_ = 0.936, *T*_max_ = 0.980
23230 measured reflections
5574 independent reflections
2632 reflections with *I* > 2σ(*I*)
*R*_int_ = 0.084

#### Refinement

*R*[*F* ^2^ > 2σ(*F* ^2^)] = 0.061
*wR*(*F* ^2^) = 0.162
*S* = 1.01
5574 reflections
301 parameters
H-atom parameters constrained
Δρ_max_ = 0.31 e Å^−3^
Δρ_min_ = −0.37 e Å^−3^

### 

Data collection: *RAPID-AUTO* (Rigaku 1998 ▶); cell refinement: *RAPID-AUTO*; data reduction: *CrystalStructure* (Rigaku/MSC, 2002 ▶); program(s) used to solve structure: *SHELXS97* (Sheldrick, 2008 ▶); program(s) used to refine structure: *SHELXL97* (Sheldrick, 2008 ▶); molecular graphics: *SHELXTL* (Sheldrick, 2008 ▶); software used to prepare material for publication: *SHELXL97*.

## Supplementary Material

Crystal structure: contains datablocks I, global. DOI: 10.1107/S1600536811012177/kp2314sup1.cif

Structure factors: contains datablocks I. DOI: 10.1107/S1600536811012177/kp2314Isup2.hkl

Additional supplementary materials:  crystallographic information; 3D view; checkCIF report

## Figures and Tables

**Table 1:** Hydrogen-bond geometry (Å, °)

*D*—H⋯*A*	*D*—H	H⋯*A*	*D*⋯*A*	*D*—H⋯*A*
C6—H6*C*⋯O1^i^	0.96	2.59	3.501 (3)	159
C19—H19*B*⋯O2^ii^	0.97	2.59	3.302 (4)	130

Symmetry codes: (i) 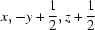; (ii) 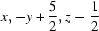.

**Fig. 1.**
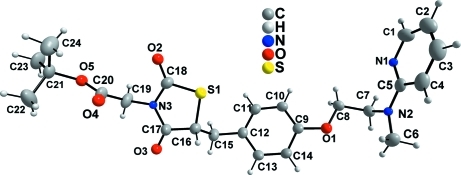
The molecular structure of the title compound, showing displacement ellipsoids at the 50% probability level for non-H atoms.

**Fig. 2.**
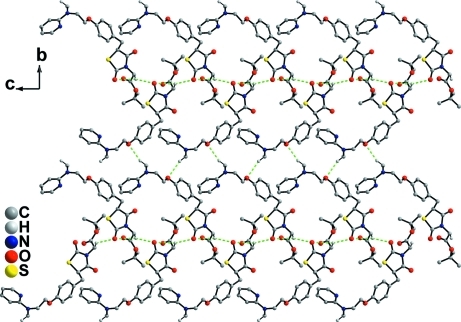
A partial packing view, showing the two-dimensional network. Dashed lines indicate the hydrogen bonds, H atoms not involved in hydrogen bonds have been omitted for clarity.

## supplementary crystallographic information

### Comment

The title compound is a type of thiazolidinedione material, which was synthesised from rosiglitazone and *tert*-butyl chloroacetate. In this paper, we report the synthesis and crystal structure of the title compound.

The title compound is chiral with a stereogenic centre C16 of the thiazolidine ring. However, it crystallises in the centrosymmetric space group as a racemate. The thiazolidine ring forms the dihedral angles of 29.22 (12) ° and 67.79 (10) ° with the benzene ring and pyridine ring, respectively. The benzene ring and the pyridine ring are tilted by 67.18 (9) ° (Fig. 1). In the crystal, intermolecular C—H···O hydrogen bonds link the molecules into a two-dimensional network (Table 1 and Fig. 2).

### Experimental

A solution of C_2_H_5_ONa (1.00 g) in anhydrous ethanol (50 mL) was added slowly to a solution of rosiglitazone (5 g) in anhydrous ethanol (125 mL) at room temperature and the mixture was stirred for 12 h. After completion of the reaction, the mixture was filtered and the white powder was dried at 323 K to afford sodium rosiglitazone (5.15 g). *tert*-Butyl chloroacetate (0.80 g) was added to a suspension of sodium rosiglitazone (1.51 g) in acetonitrile (70 mL) and the mixture was refluxed for 11 h. After completion of the reaction, the warm suspension was filtered and the filtrates were concentrated under vacuum. Single crystals were obtained by recrystallisation from EtOH : EtOAc (1 : 2) at room temperature.

### Refinement

All H atoms, were positioned geometrically with C—H distances ranging from 0.93 Å to 0.97Å and refined as riding on their parent atoms with *U*_iso_(H) = 1.5*U*_eq_(C)

### Crystal data

**Table:**

C_24_H_29_N_3_O_5_S	*F*(000) = 1000
*M**_r_* = 471.56	*D*_x_ = 1.263 Mg m^−^^3^
Monoclinic, *P*2_1_/*c*	Mo *K*α radiation, λ = 0.71073 Å
Hall symbol: -P 2ybc	Cell parameters from 10831 reflections
*a* = 25.621 (5) Å	θ = 3.1–27.5°
*b* = 9.886 (2) Å	µ = 0.17 mm^−^^1^
*c* = 9.874 (2) Å	*T* = 291 K
β = 97.32 (3)°	Rod, colorless
*V* = 2480.6 (9) Å^3^	0.40 × 0.13 × 0.12 mm
*Z* = 4	

### Data collection

**Table:**

Rigaku R-AXIS RAPID diffractometer	5574 independent reflections
Radiation source: fine-focus sealed tube	2632 reflections with *I* > 2σ(*I*)
graphite	*R*_int_ = 0.084
ω scan	θ_max_ = 27.5°, θ_min_ = 3.1°
Absorption correction: multi-scan (*ABSCOR*; Higashi, 1995)	*h* = −33→33
*T*_min_ = 0.936, *T*_max_ = 0.980	*k* = −12→12
23230 measured reflections	*l* = −11→12

### Refinement

**Table:**

Refinement on *F*^2^	Primary atom site location: structure-invariant direct methods
Least-squares matrix: full	Secondary atom site location: difference Fourier map
*R*[*F*^2^ > 2σ(*F*^2^)] = 0.061	Hydrogen site location: inferred from neighbouring sites
*wR*(*F*^2^) = 0.162	H-atom parameters constrained
*S* = 1.01	*w* = 1/[σ^2^(*F*_o_^2^) + (0.0701*P*)^2^] where *P* = (*F*_o_^2^ + 2*F*_c_^2^)/3
5574 reflections	(Δ/σ)_max_ < 0.001
301 parameters	Δρ_max_ = 0.31 e Å^−^^3^
0 restraints	Δρ_min_ = −0.37 e Å^−^^3^

### Special details

**Table:**

Geometry. All esds (except the esd in the dihedral angle between two l.s. planes) are estimated using the full covariance matrix. The cell esds are taken into account individually in the estimation of esds in distances, angles and torsion angles; correlations between esds in cell parameters are only used when they are defined by crystal symmetry. An approximate (isotropic) treatment of cell esds is used for estimating esds involving l.s. planes.
Refinement. Refinement of F^2^ against ALL reflections. The weighted R-factor wR and goodness of fit S are based on F^2^, conventional R-factors R are based on F, with F set to zero for negative F^2^. The threshold expression of F^2^ > 2sigma(F^2^) is used only for calculating R-factors(gt) etc. and is not relevant to the choice of reflections for refinement. R-factors based on F^2^ are statistically about twice as large as those based on F, and R- factors based on ALL data will be even larger.

### Fractional atomic coordinates and isotropic or equivalent isotropic displacement parameters (Å^2^)

**Table:**

	*x*	*y*	*z*	*U*_iso_*/*U*_eq_	
C1	0.48020 (14)	0.6375 (3)	0.8137 (3)	0.0804 (9)	
H1	0.5087	0.6933	0.8041	0.097*	
C2	0.45977 (18)	0.6428 (4)	0.9329 (4)	0.0888 (11)	
H2	0.4740	0.6990	1.0037	0.107*	
C3	0.41744 (19)	0.5625 (4)	0.9454 (3)	0.0924 (12)	
H3	0.4020	0.5643	1.0257	0.111*	
C4	0.39732 (14)	0.4788 (3)	0.8404 (3)	0.0754 (9)	
H4	0.3681	0.4248	0.8481	0.090*	
C5	0.42170 (11)	0.4768 (3)	0.7222 (3)	0.0564 (7)	
C6	0.35989 (14)	0.3066 (3)	0.6164 (3)	0.0863 (10)	
H6A	0.3548	0.2545	0.5337	0.129*	
H6B	0.3294	0.3618	0.6225	0.129*	
H6C	0.3651	0.2466	0.6934	0.129*	
C7	0.42961 (11)	0.4002 (3)	0.4911 (3)	0.0596 (7)	
H7A	0.4261	0.3132	0.4455	0.072*	
H7B	0.4669	0.4185	0.5142	0.072*	
C8	0.40612 (10)	0.5069 (3)	0.3943 (3)	0.0553 (7)	
H8A	0.4049	0.5928	0.4413	0.066*	
H8B	0.4271	0.5180	0.3201	0.066*	
C9	0.32627 (10)	0.5409 (2)	0.2430 (2)	0.0485 (6)	
C10	0.34080 (11)	0.6687 (2)	0.2067 (3)	0.0528 (7)	
H10	0.3712	0.7089	0.2507	0.063*	
C11	0.30955 (11)	0.7364 (3)	0.1040 (3)	0.0586 (7)	
H11	0.3195	0.8226	0.0794	0.070*	
C12	0.26421 (11)	0.6808 (3)	0.0368 (3)	0.0543 (7)	
C13	0.25053 (11)	0.5518 (3)	0.0757 (3)	0.0592 (7)	
H13	0.2201	0.5116	0.0322	0.071*	
C14	0.28120 (11)	0.4822 (3)	0.1775 (3)	0.0570 (7)	
H14	0.2715	0.3958	0.2019	0.068*	
C15	0.23076 (12)	0.7571 (3)	−0.0749 (3)	0.0651 (8)	
H15A	0.2124	0.6925	−0.1378	0.078*	
H15B	0.2536	0.8104	−0.1253	0.078*	
C16	0.19073 (11)	0.8502 (3)	−0.0224 (3)	0.0579 (7)	
H16	0.1655	0.7957	0.0207	0.070*	
C17	0.16154 (12)	0.9282 (3)	−0.1407 (3)	0.0644 (8)	
C18	0.19831 (12)	1.1093 (3)	−0.0082 (3)	0.0621 (8)	
C19	0.14767 (14)	1.1623 (3)	−0.2281 (3)	0.0773 (10)	
H19A	0.1337	1.1146	−0.3106	0.093*	
H19B	0.1758	1.2207	−0.2502	0.093*	
C20	0.10508 (13)	1.2475 (3)	−0.1812 (3)	0.0659 (8)	
C21	0.05990 (12)	1.4630 (3)	−0.2386 (3)	0.0626 (8)	
C22	0.00677 (15)	1.4008 (4)	−0.2715 (5)	0.1199 (15)	
H22A	0.0054	1.3502	−0.3548	0.180*	
H22B	−0.0194	1.4708	−0.2823	0.180*	
H22C	0.0001	1.3416	−0.1987	0.180*	
C23	0.06953 (16)	1.5643 (3)	−0.3460 (3)	0.0941 (11)	
H23A	0.1036	1.6043	−0.3229	0.141*	
H23B	0.0431	1.6335	−0.3510	0.141*	
H23C	0.0680	1.5198	−0.4329	0.141*	
C24	0.06951 (18)	1.5227 (4)	−0.0975 (4)	0.1122 (14)	
H24A	0.0647	1.4541	−0.0313	0.168*	
H24B	0.0451	1.5951	−0.0899	0.168*	
H24C	0.1048	1.5569	−0.0811	0.168*	
N1	0.46247 (10)	0.5579 (3)	0.7084 (2)	0.0678 (7)	
N2	0.40565 (10)	0.3921 (2)	0.6158 (2)	0.0644 (6)	
N3	0.16884 (9)	1.0652 (2)	−0.1261 (2)	0.0588 (6)	
O1	0.35434 (7)	0.46396 (17)	0.34286 (17)	0.0591 (5)	
O2	0.20758 (10)	1.2268 (2)	0.0164 (2)	0.0896 (7)	
O3	0.13683 (10)	0.8764 (2)	−0.2388 (2)	0.0971 (8)	
O4	0.07940 (10)	1.2150 (2)	−0.0940 (3)	0.1076 (9)	
O5	0.10097 (8)	1.36116 (17)	−0.25172 (18)	0.0631 (5)	
S1	0.21978 (3)	0.97432 (8)	0.09819 (7)	0.0724 (3)	

### Atomic displacement parameters (Å^2^)

**Table:**

	*U*^11^	*U*^22^	*U*^33^	*U*^12^	*U*^13^	*U*^23^
C1	0.079 (3)	0.076 (2)	0.083 (2)	−0.0036 (18)	−0.0055 (19)	−0.0139 (18)
C2	0.108 (3)	0.082 (2)	0.071 (2)	0.023 (2)	−0.006 (2)	−0.0150 (19)
C3	0.119 (3)	0.103 (3)	0.059 (2)	0.039 (3)	0.027 (2)	0.004 (2)
C4	0.081 (2)	0.080 (2)	0.0676 (19)	0.0148 (18)	0.0219 (18)	0.0188 (17)
C5	0.0554 (18)	0.0565 (16)	0.0555 (16)	0.0087 (14)	0.0001 (13)	0.0145 (13)
C6	0.080 (2)	0.083 (2)	0.093 (2)	−0.027 (2)	−0.0012 (18)	0.0209 (18)
C7	0.0552 (18)	0.0625 (17)	0.0587 (16)	0.0017 (14)	−0.0017 (14)	−0.0014 (13)
C8	0.0478 (17)	0.0593 (16)	0.0578 (15)	−0.0049 (13)	0.0025 (13)	−0.0015 (13)
C9	0.0478 (16)	0.0450 (14)	0.0531 (14)	0.0036 (12)	0.0084 (12)	−0.0045 (12)
C10	0.0476 (17)	0.0481 (15)	0.0627 (16)	−0.0029 (13)	0.0069 (13)	−0.0044 (13)
C11	0.063 (2)	0.0463 (15)	0.0677 (17)	0.0011 (14)	0.0115 (15)	0.0017 (13)
C12	0.0566 (18)	0.0488 (15)	0.0574 (16)	0.0090 (14)	0.0064 (14)	−0.0015 (12)
C13	0.0552 (18)	0.0460 (15)	0.0729 (17)	0.0014 (13)	−0.0052 (14)	−0.0078 (13)
C14	0.0558 (18)	0.0393 (13)	0.0738 (17)	−0.0004 (13)	0.0001 (14)	−0.0012 (13)
C15	0.070 (2)	0.0600 (17)	0.0648 (16)	0.0129 (15)	0.0057 (15)	0.0017 (14)
C16	0.0604 (19)	0.0500 (15)	0.0625 (16)	0.0020 (13)	0.0047 (14)	0.0025 (13)
C17	0.065 (2)	0.0519 (16)	0.0727 (19)	0.0055 (15)	−0.0069 (16)	0.0018 (15)
C18	0.069 (2)	0.0494 (17)	0.0690 (18)	0.0050 (15)	0.0149 (16)	−0.0065 (13)
C19	0.105 (3)	0.0603 (18)	0.0681 (18)	0.0257 (18)	0.0189 (18)	0.0154 (15)
C20	0.072 (2)	0.0533 (17)	0.0719 (18)	0.0089 (15)	0.0084 (17)	0.0161 (15)
C21	0.062 (2)	0.0532 (16)	0.0734 (18)	0.0148 (15)	0.0103 (15)	0.0081 (14)
C22	0.063 (3)	0.107 (3)	0.181 (4)	−0.002 (2)	−0.015 (3)	0.037 (3)
C23	0.109 (3)	0.065 (2)	0.111 (3)	0.026 (2)	0.025 (2)	0.0304 (19)
C24	0.143 (4)	0.104 (3)	0.088 (2)	0.043 (3)	0.011 (2)	−0.012 (2)
N1	0.0619 (17)	0.0731 (16)	0.0675 (15)	−0.0104 (14)	0.0039 (12)	−0.0074 (13)
N2	0.0641 (16)	0.0662 (15)	0.0616 (14)	−0.0177 (13)	0.0031 (12)	0.0030 (12)
N3	0.0716 (17)	0.0434 (12)	0.0600 (13)	0.0119 (11)	0.0029 (12)	0.0051 (11)
O1	0.0539 (12)	0.0533 (10)	0.0666 (11)	−0.0021 (9)	−0.0060 (9)	0.0092 (9)
O2	0.1121 (19)	0.0509 (12)	0.1059 (16)	−0.0064 (12)	0.0145 (14)	−0.0160 (12)
O3	0.103 (2)	0.0739 (15)	0.0992 (16)	0.0017 (13)	−0.0450 (14)	−0.0055 (13)
O4	0.106 (2)	0.0959 (17)	0.131 (2)	0.0322 (15)	0.0555 (17)	0.0592 (16)
O5	0.0707 (14)	0.0510 (10)	0.0696 (11)	0.0146 (10)	0.0165 (10)	0.0144 (9)
S1	0.0846 (6)	0.0628 (5)	0.0646 (5)	0.0150 (4)	−0.0102 (4)	−0.0077 (4)

### Geometric parameters (Å, °)

**Table:**

C1—N1	1.337 (4)	C13—H13	0.9300
C1—C2	1.349 (5)	C14—H14	0.9300
C1—H1	0.9300	C15—C16	1.517 (4)
C2—C3	1.363 (5)	C15—H15A	0.9700
C2—H2	0.9300	C15—H15B	0.9700
C3—C4	1.374 (5)	C16—C17	1.515 (4)
C3—H3	0.9300	C16—S1	1.803 (3)
C4—C5	1.392 (4)	C16—H16	0.9800
C4—H4	0.9300	C17—O3	1.202 (3)
C5—N1	1.337 (3)	C17—N3	1.373 (4)
C5—N2	1.365 (3)	C18—O2	1.204 (3)
C6—N2	1.446 (4)	C18—N3	1.375 (4)
C6—H6A	0.9600	C18—S1	1.744 (3)
C6—H6B	0.9600	C19—N3	1.446 (3)
C6—H6C	0.9600	C19—C20	1.498 (4)
C7—N2	1.446 (3)	C19—H19A	0.9700
C7—C8	1.497 (3)	C19—H19B	0.9700
C7—H7A	0.9700	C20—O4	1.193 (3)
C7—H7B	0.9700	C20—O5	1.319 (3)
C8—O1	1.424 (3)	C21—O5	1.474 (3)
C8—H8A	0.9700	C21—C22	1.491 (4)
C8—H8B	0.9700	C21—C23	1.501 (4)
C9—O1	1.374 (3)	C21—C24	1.505 (4)
C9—C14	1.377 (4)	C22—H22A	0.9600
C9—C10	1.378 (3)	C22—H22B	0.9600
C10—C11	1.382 (4)	C22—H22C	0.9600
C10—H10	0.9300	C23—H23A	0.9600
C11—C12	1.377 (4)	C23—H23B	0.9600
C11—H11	0.9300	C23—H23C	0.9600
C12—C13	1.390 (4)	C24—H24A	0.9600
C12—C15	1.509 (4)	C24—H24B	0.9600
C13—C14	1.378 (4)	C24—H24C	0.9600
			
N1—C1—C2	124.8 (4)	H15A—C15—H15B	107.7
N1—C1—H1	117.6	C17—C16—C15	109.5 (2)
C2—C1—H1	117.6	C17—C16—S1	106.53 (18)
C1—C2—C3	117.2 (3)	C15—C16—S1	113.5 (2)
C1—C2—H2	121.4	C17—C16—H16	109.1
C3—C2—H2	121.4	C15—C16—H16	109.1
C2—C3—C4	120.6 (3)	S1—C16—H16	109.1
C2—C3—H3	119.7	O3—C17—N3	123.8 (3)
C4—C3—H3	119.7	O3—C17—C16	124.2 (3)
C3—C4—C5	118.4 (3)	N3—C17—C16	111.9 (2)
C3—C4—H4	120.8	O2—C18—N3	123.4 (3)
C5—C4—H4	120.8	O2—C18—S1	125.3 (3)
N1—C5—N2	116.8 (2)	N3—C18—S1	111.37 (19)
N1—C5—C4	121.1 (3)	N3—C19—C20	112.4 (2)
N2—C5—C4	122.0 (3)	N3—C19—H19A	109.1
N2—C6—H6A	109.5	C20—C19—H19A	109.1
N2—C6—H6B	109.5	N3—C19—H19B	109.1
H6A—C6—H6B	109.5	C20—C19—H19B	109.1
N2—C6—H6C	109.5	H19A—C19—H19B	107.9
H6A—C6—H6C	109.5	O4—C20—O5	126.6 (3)
H6B—C6—H6C	109.5	O4—C20—C19	124.2 (3)
N2—C7—C8	113.8 (2)	O5—C20—C19	109.1 (3)
N2—C7—H7A	108.8	O5—C21—C22	110.0 (3)
C8—C7—H7A	108.8	O5—C21—C23	102.1 (2)
N2—C7—H7B	108.8	C22—C21—C23	110.4 (3)
C8—C7—H7B	108.8	O5—C21—C24	108.4 (3)
H7A—C7—H7B	107.7	C22—C21—C24	113.6 (3)
O1—C8—C7	107.4 (2)	C23—C21—C24	111.7 (3)
O1—C8—H8A	110.2	C21—C22—H22A	109.5
C7—C8—H8A	110.2	C21—C22—H22B	109.5
O1—C8—H8B	110.2	H22A—C22—H22B	109.5
C7—C8—H8B	110.2	C21—C22—H22C	109.5
H8A—C8—H8B	108.5	H22A—C22—H22C	109.5
O1—C9—C14	115.8 (2)	H22B—C22—H22C	109.5
O1—C9—C10	124.1 (2)	C21—C23—H23A	109.5
C14—C9—C10	120.1 (2)	C21—C23—H23B	109.5
C9—C10—C11	119.0 (3)	H23A—C23—H23B	109.5
C9—C10—H10	120.5	C21—C23—H23C	109.5
C11—C10—H10	120.5	H23A—C23—H23C	109.5
C12—C11—C10	122.3 (3)	H23B—C23—H23C	109.5
C12—C11—H11	118.8	C21—C24—H24A	109.5
C10—C11—H11	118.8	C21—C24—H24B	109.5
C11—C12—C13	117.4 (2)	H24A—C24—H24B	109.5
C11—C12—C15	121.2 (3)	C21—C24—H24C	109.5
C13—C12—C15	121.4 (3)	H24A—C24—H24C	109.5
C14—C13—C12	121.3 (3)	H24B—C24—H24C	109.5
C14—C13—H13	119.4	C1—N1—C5	117.8 (3)
C12—C13—H13	119.4	C5—N2—C7	120.2 (2)
C9—C14—C13	119.9 (2)	C5—N2—C6	121.4 (3)
C9—C14—H14	120.0	C7—N2—C6	117.9 (2)
C13—C14—H14	120.0	C17—N3—C18	117.1 (2)
C12—C15—C16	113.5 (2)	C17—N3—C19	123.2 (2)
C12—C15—H15A	108.9	C18—N3—C19	119.7 (2)
C16—C15—H15A	108.9	C9—O1—C8	118.04 (19)
C12—C15—H15B	108.9	C20—O5—C21	123.1 (2)
C16—C15—H15B	108.9	C18—S1—C16	92.91 (13)

### Hydrogen-bond geometry (Å, °)

**Table:**

*D*—H···*A*	*D*—H	H···*A*	*D*···*A*	*D*—H···*A*
C6—H6C···O1^i^	0.96	2.59	3.501 (3)	159
C19—H19B···O2^ii^	0.97	2.59	3.302 (4)	130

Symmetry codes: (i) *x*, −*y*+1/2, *z*+1/2; (ii) *x*, −*y*+5/2, *z*−1/2.
